# The association between anemia and postpartum depression: A systematic review and meta-analysis

**DOI:** 10.22088/cjim.10.2.115

**Published:** 2019

**Authors:** Milad Azami, Gholamreza Badfar, Zahra Khalighi, Parvin Qasemi, Masoumeh Shohani, Ali Soleymani, Shamsi Abbasalizadeh

**Affiliations:** 1 Faculty of Medicine, Ilam University of Medical Sciences, Ilam, Iran; 2 Women’s Reproductive Health Research Center, School of Medicine, Tabriz University of Medical Sciences, Tabriz, Iran; 3 Department of Pediatrics, Behbahan Faculty of Medical Sciences, Behbahan, Iran; 4 Biotechnology and Medicinal Plants Research Center, Ilam University of Medical Sciences, Ilam, Iran; 5 Faculty of Allied Medical Sciences, Ilam University of Medical Sciences, Ilam, Iran; 6 Department of Nursing, Faculty of Nursing and Midwifery, Ilam University of Medical Sciences, Ilam, Iran; 7 Dezful University of Medical Sciences, Dezful, Iran

**Keywords:** Anemia, Postpartum Depression, Meta-Analysis.

## Abstract

**Background:**

The association between anemia and postpartum depression (PPD) has been reported to be controversial in different studies. Therefore, this study aimed to provide a comprehensive assessment of anemia and PPD.

**Methods:**

This review study was conducted according to the MOOSE protocol and results have been reported according to the PRISMA guideline. We searched epidemiologic studies published until January 2018 in nine English databases including Scopus, PubMed/Medline, Science Direct, Embase, Web of Science, CINAHL, Cochrane Library, EBSCO and Google Scholar using English MeSH keywords. The heterogeneity of the studies was assessed using the Cochran’s Q test and I^2^ index. Data were analyzed using a random effects model and comprehensive meta-analysis (CMA) software version 2.

**Results:**

In the 10 studies, the association between postpartum anemia and PPD was significant (heterogeneity test: P<0.001, I^2^=74.62%), and RR=1.887 (95%CI: 1.255-2.838, P=0.002). In 8 studies, anemia during pregnancy significantly increased the risk of postpartum depression (heterogeneity test: P=0.116, I^2^=36.422%), RR=1.240 (1.001-1.536, P=0.048). The subgroup analysis of postpartum anemia and PPD was not significant for the variables of quality of studies, study design, and the period of evaluating depression and anemia. The subgroup analysis of anemia during pregnancy and PPD was not significant for the period of evaluating depression. Publication bias did not affect the results of the studies.

**Conclusion:**

Meta-analysis results showed anemia during pregnancy and after pregnancy that significantly increased the risk of postpartum depression. Therefore, prevention, identification and treatment of anemia in pregnant women seem necessary.

Anemia is one of the most important public health issues worldwide, which has a great impact on the physical, mental ability of people at work, and is the most common type of iron-deficiency anemia (1); about 80% of non-physiologic anemia during pregnancy occurs due to iron deficiency (2). Therefore, paying attention to the nutritional status of women during pregnancy is very important. The prevalence of anemia in pregnant women is affected by geographical region, lifestyle, and diet, and is reported to be between 14-80% in different societies (3-5). The effect of anemia on the adverse pregnancy outcomes, including pre-eclampsia, premature rupture of membranes, low birth weight, preterm birth, fetal and maternal deaths have been shown in various studies (6-8). Depression during pregnancy and PPD are one of the most common problems in pregnant women (9).

The cause of this problem is associated with genetics, history of mental illness, and physiological, psychological and social changes, hormonal changes, physical discomfort such as nausea and vomiting, fatigue, sudden pain during pregnancy, and is also associated with the health of the fetus and even the mode of delivery (10). Depression has short-term and long-term effects on mother and fetus, including high-risk behaviors, preeclampsia, negative pregnancy outcomes such as low birth weight, prematurity, small head circumference and increased PPD (11-14). Additionally, depression during pregnancy is also related to a range of other negative outcomes such as social isolation (10), marital conflicts (15), delayed motor skills or intellectual development in the infant (16), embryonic growth restriction, and high stress response in newborn at delivery (17, 18). Various studies have investigated the association between anemia and PPD, and the results of these studies are diverse (19-23) and no meta-analysis on this topic is available. Thus, a systematic review and meta-analysis seems necessary.

Obviously, in meta-analytical methods, by collecting data from several studies, the number of samples is greater and therefore, the range of variations and probabilities is reduced. As a result, the significance of statistical findings increases (24-27). On the other hand, the motto of the World Health Organization in 2017 (Depression: Let's Talk) emphasizes global attention to the issue of depression. The present study aimed to investigate the association between anemia and PPD.

## Methods

**Study protocol** **:** This review study was conducted on the basis of the Meta-analysis of observational studies in epidemiology (MOOSE) protocol and results were reported according to the Preferred Reporting Items for Systematic Reviews and Meta-Analyses (PRISMA) guideline for systematic review and meta-analysis (27). All the steps of study were taken by two researchers independently. In cases of disagreement, a third researcher helped reach an agreement. 

**Search strategy** **:** We searched epidemiologic studies published until January 2018 without time limit in nine English databases including Scopus, PubMed/Medline, Science Direct, Embase, Web of Science (ISI), CINAHL, Cochrane Library, EBSCO and Google Scholar search engine using English MeSH keywords: Anemia, Anaemia, Hemoglobin, Ferritin, Pregnancy, Pregnant Woman, Prenatal Care, Complications of Pregnancy, Postpartum Depression, Mental Disorders, and Mental Health. A combination of words was used with functions “AND” and “OR”. An example of the PubMed search strategy is shown in Appendix 1. A manual search was also done using the reference list of articles searched on the above-mentioned websites. 

**Inclusion and exclusion criteria** **: **Inclusion criteria according to PICO (related to evidence-based medicine) (28): 1) **P**opulation: epidemiologic studies (cross-sectional, cohort and case studies) that investigated the association between anemia during pregnancy or postpartum anemia and PPD; 2) **I**ntervention: hematological test to confirm anemia and questionnaire to confirm PPD; 3) **C**omparison: That shows the rate of PPD prevalence in anemic patients compared to non-anemic patients; 4) **O**utcome: Estimate the association between anemia and PPD. 

Exclusion criteria were: 1) sample size other than pregnant women or postpartum women; 2) sample size with a history of mental illness or use of antidepressants; 3) letters to the Editor without original data, review and case report and 4) duplicate studies.

**Qualitative assessment** **:** The modified Newcastle Ottawa Scale (NOS) for cross-sectional studies (29) was used to assess the quality of the studies. The quality of the studies was divided into three categories: unsatisfactory (less than 5 points), satisfactory (5-6 points) and good or very good (7-10 points). Finally, the points given to the articles were compared by two researchers and a general discussion was carried out in cases of disagreement. The minimum score for entering the meta-analysis process was 5.

**Data extraction** **:** For data extraction, a premade checklist, including the name of the author (s), year of study, place of study, study design, sample size (total, case and control), mean and SD (standard deviation) for hemoglobin in case and control groups, P-value for correlation, age (mean±SD), gestational age (mean±SD), anemia cut-off point, depression diagnostic tool, depression cut-off point, and odds ratio (OR) or relative risk (RR) with 95% confidence interval (CI) was used.

**Grading of evidence:** We categorized the overall methodological quality of each distributed analysis using the Grading of Recommendations Assessment, Development and Evaluation (short GRADE), while taking into account study limitations (risk of bias), inconsistency, imprecision, and indirectness, and publication bias (30). Then, the quality of evidence was divided into high, moderate, low or very low.

**Statistical analysis** **:** The results of the study were analyzed using Comprehensive Meta-Analysis (CMA) Version 2. For calculating RR and 95% CI, we used: 1) Event rate and total sample size for each of the groups (case and control) in the studies of Parhizkar (31), Akbari (32), Eckerdal (33), Paterson (34); 2) P-value and sample size for correlation in the studies of Goshtasebi (20) and Corwin (21); 3) Mean, SD and sample size for each of the groups in the study of Armony (22); and 4) in the study of Alharbi (23), RR and 95% CI were reported. Finally, the RR_S_ and 95% CI were combined for meta-analysis. The heterogeneity of the studies was assessed using the Cochran’s Q test and I^2^ index. In this regard, interpretation was as follows: (0-24% may not be important, 25-49% may indicate moderate heterogeneity, 50-75% indicate substantial heterogeneity and over 75% indicate considerable heterogeneity) (35). Therefore, we observed statistical heterogeneity, and thus, random effects model was used (36). A subgroup analysis was conducted to find out the cause of heterogeneity. Sensitivity analysis was also performed. The Begg and Egger’s tests were used to assess the publication bias. The significance level of the test was considered to be p<0.05.

## Results

**Figure 1:**
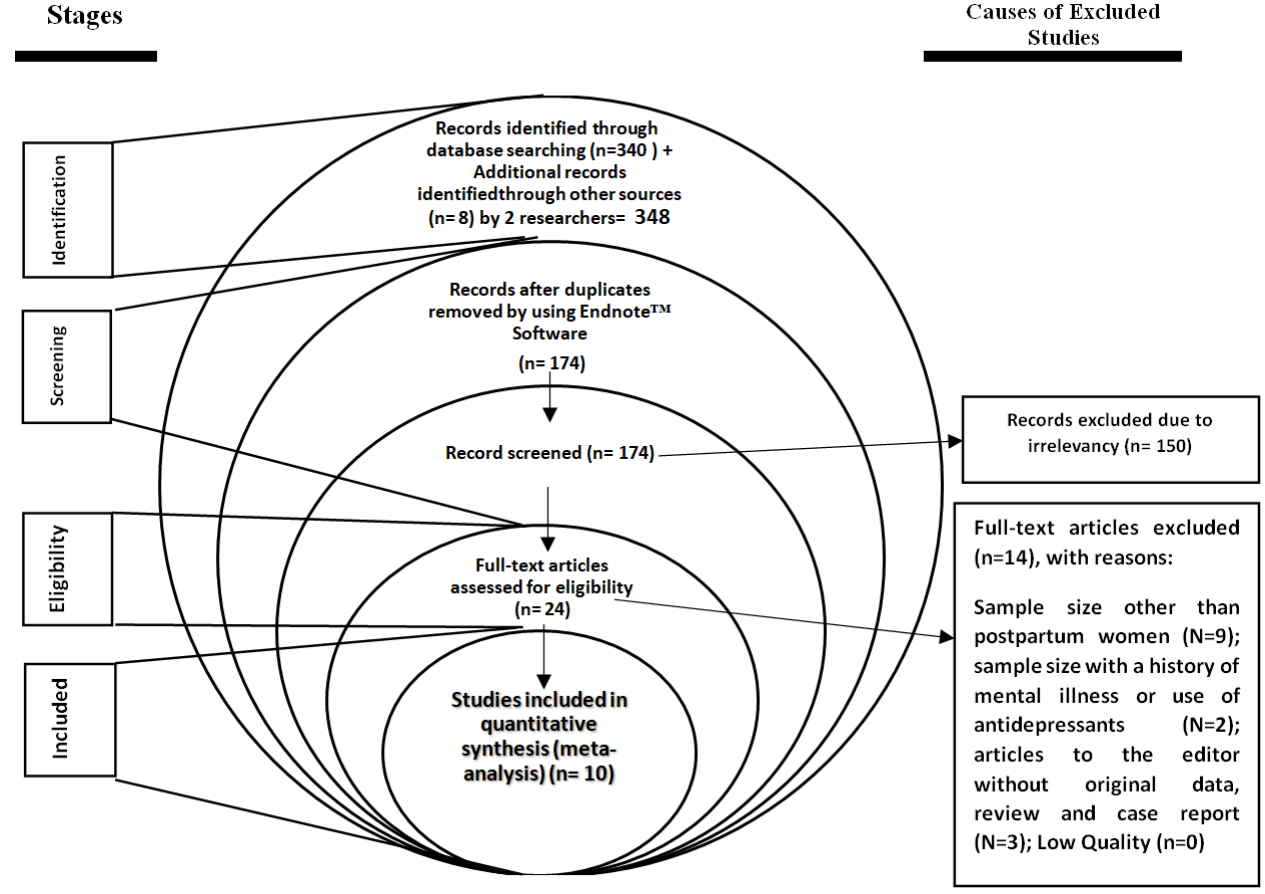
PRISMA flowchart

**Study characteristics and methodological quality** **:** The process of study selection is shown in figure 1. In this systematic study, 348 articles were identified based on an initial search by two researchers. 174 duplicate studies were excluded. 150 irrelevant studies were excluded. After final evaluation and review, 14 studies were excluded due to: sample size other than pregnant women or postpartum women (N=9); sample size with a history of mental illness or the use of antidepressants (N=2); letters to the editor without original data, reviews and case reports (N=3). Finally, 10 studies [10 studies for postpartum anemia and PD and 8 studies for anemia during pregnancy and PPD] entered the quantitative meta-analysis process. The mean age of the participants in the study was 28.16 years (95% CI: 25.30 - 31.03). Other characteristics of studies are summarized in table 1.

**Table 1:** Summary characteristics of studies entered into the meta-analysis

**Ref**	**First author, Published year**	**Survey**	**Design**	**Place**	**Sample size**		**Tool for ** ** depression**	**Time point of**		**Cut-off point**		**RR/ OR** *	**(95%CI)** *		**Quality**
					**Case**	**Control**		**postpartum depression**	**Anemia**	**depression**	**anemia**		**Lower**	**Upper**	
(20)	Goshtasebi, 2013	1	Cohort	Iran	14	240	EPDS	At 4-6 W	4-6 W postpartum	13	11	3.746	1.178	11.906	Satisfactory
(21)	Corwin, 2003	1	Cohort	America	8	29	CES-D	At 4 W	At 7 and 14 days postpartum	16	12	5.846	1.337	25.56	Satisfactory
(22)	Armony, 2012	1	Cohort	China	140	124	EPDS	At 24–48 h	At 3th days postpartum	10	11	1.0	0.645	1.55	Good
(22)	Armony, 2012^**^	1	Cohort	China	130	118	EPDS	At 6 W	At 3 days postpartum	10	11	0.797	0.507	1.253	Good
(31)	Parhizkar, 2012	1	Cross sectional	Iran	316	84	EPDS	At 4 W	At 7th day postpartum	10	12	2.016	1.23	3.305	Satisfactory
(23)	Alharbi, 2014	1	Cohort	Saudi Arabia	166	186	EPDS	8- 12 W	At 8–12 W postpartum	10	11	1.7	1.05	2.74	Good
(32)	Akbari, 2008	1	Cohort	Iran	28	81	EPDS	At 4 W	At 7th postpartum day	10	11	3.22	1.494	6.941	Good
(32)	Akbari, 2008	1	Cohort	Iran	29	96	EPDS	At 4 W	At 4 W postpartum	10	11	6.591	2.962	14.667	Good
(33)	Eckerdal, 2016	2	Cohort	Sweden	106	340	EPDS	At 6 W	At 6-8 W postpartum	12	11	1.107	0.371	3.296	Good
(34)	Paterson, 1994	1	Cross sectional	United kingdom	251	598	EPDS	At 10th day	At 10th day postpartum	14	10.5	1.331	0.791	2.241	Satisfactory
(22)	Armony, 2012	2	Cohort	China	12	125	EPDS	At 6 W	Early/mid pregnancy	10	11	1.278	0.436	3.744	Good
(22)	Armony, 2012	2	Cohort	China	54	81	EPDS	6 W	Late pregnancy	10	11	1.278	0.684	2.389	Good
(22)	Armony, 2012	2	Cohort	China	72	483	EPDS	At 24–48 h	Early/mid pregnancy	10	11	0.919	0.584	1.439	Good
(22)	Armony, 2012	2	Cohort	China	71	417	EPDS	At 6 W	Early/mid pregnancy	10	11	0.736	0.466	1.162	Good
(22)	Armony, 2012	2	Cohort	China	181	366	EPDS	At 24–48 h	Late pregnancy	10	11	1.35	0.977	1.865	Good
(22)	Armony, 2012	2	Cohort	China	165	315	EPDS	At 6 W	Late pregnancy	10	11	1.247	0.886	1.756	Good
(32)	Akbari, 2008	1	Cohort	Iran			EPDS	At 4 W	38-40 W pregnancy	10	11	1.8	1.2	2.7	Good
(33)	Eckerdal, 2016	1	Cohort	Sweden	31	412	EPDS	At 6 W	During pregnancy	11	12	1.782	0.963	3.3	Good

1: Postpartum anemia on postpartum depression; 2: Anemia during pregnancy and postpartum depression. RR; Relative risk, OR; Odds ratio, CI: Confidence interval, W; week, H; hours,

* RR/ OR and 95% CI was estimated.

** Repetitive studies have been included and estimate the relation between anemia and postpartum depression for more than survey (anemia during pregnancy or postpartum anemia), more than one sample size or also different time point of postpartum depression or anemia.

**Figure 2:**
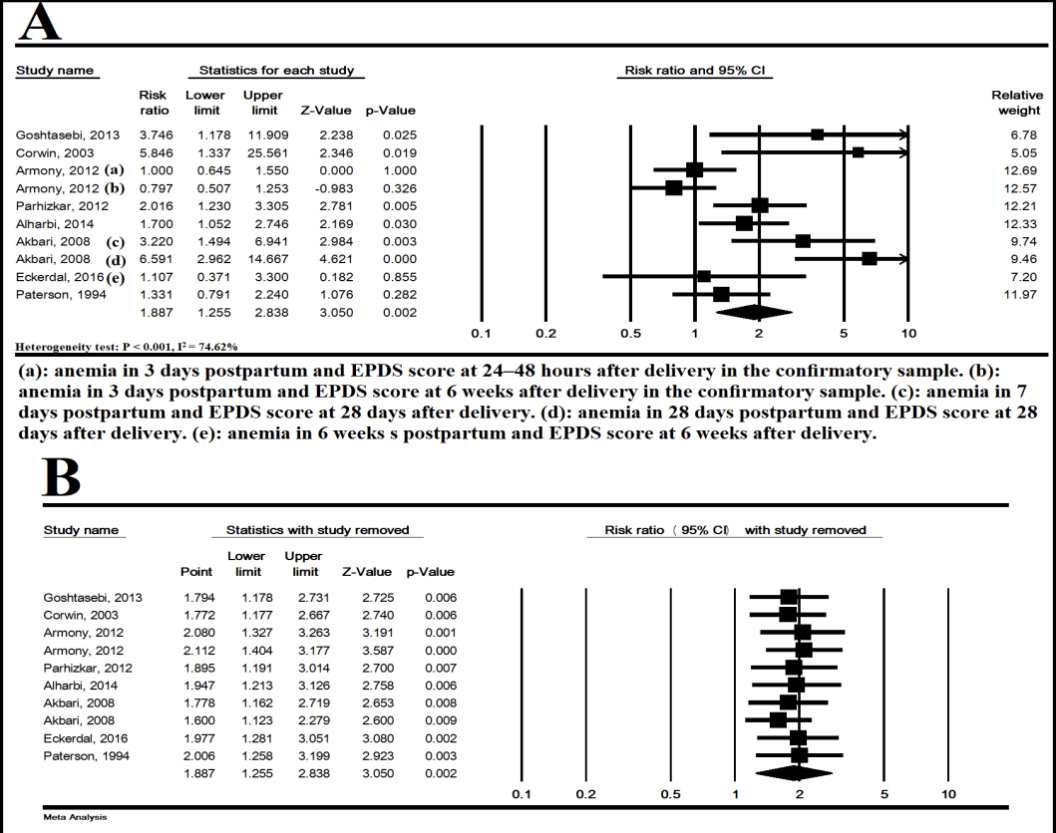
The association of postpartum anemia and postpartum depression (A) and sensitivity analysis with removed one study (B). Random effects model. CI; Confidence interval

**T** **he association between postpartum anemia** **and** **PPD:** In 10 studies, PPD was significantly higher in anemic women than non-anemic women based on the random effects model (heterogeneity test: P< 0.001, I^2^^=^ 74.62%), and RR= 1.887 (95% CI: 1.255-2.838, P= 0.002) (figure 2-A). In figure 2-B, this association is shown by omitting one study at a time, and the results showed that the overall estimate was strong (sensitivity analysis).

**Figure 3:**
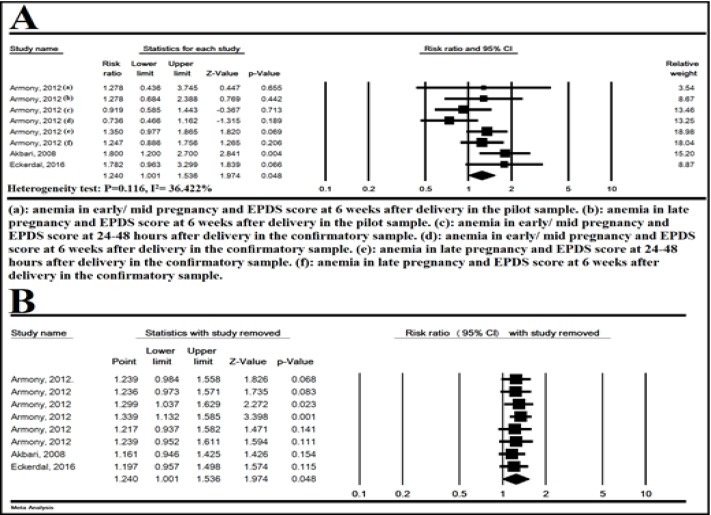
The association of anemia during pregnancy and postpartum depression (A) and sensitivity with one study removed analysis (B), Random effects model. CI: Confidence interval

**The association between anemia during pregnancy and PPD** **:** In 8 studies, anemia during pregnancy significantly increased the risk of PPD (heterogeneity test: P= 0.116, I^2^ = 36.422%), RR= 1.240 (95% CI: 1.001-1.536, P= 0.048) (figure 2). Figure 3-B shows the sensitivity analysis and the results showed that the overall estimate was strong.

**Subgroup analysis:** The subgroup analysis of postpartum anemia and PPD was not significant for the variables of geographic regions (P=0.113), study design (P=0.545), quality of the studies (P=0.604), and the period of evaluating depression (P=0.604) and anemia (P= 0.261) (table 2). The subgroup analysis of anemia during pregnancy and PPD was not significant for the period of evaluating depression (P= 0.588) (table 2).

**Table 2:** Subgroup analysis of anemia and postpartum depression

**PValue**	**RR**	**95% CI**	**Heterogeneity**				**Study(N)**	**Variable**		
			**I** ^2^ ** (%)**	**PValue**	**df**	**Q**				
0.009	1.973	1.185-3.285	81.054	< 0.001	6	31.668	7	Asia	Regions	Postpartum anemia
0.019	5.846	1.337-25.561	0	-	0	0	1	America		
0.294	1.286	0.804-2.058	0	0.765	1	0.089	2	Europe		
Test for subgroup differences: Q= 4.366, df(Q)= 2, P= 0.113										
0.10	2.038	1.182-3.514	79.399	< 0.001	7	33.979	8	Cohort	Study design	
0.016	1.652	1.100-2.481	22.172	22.172	1	1.285	2	Cross sectional		
Test for subgroup differences: Q= 0.366, df(Q)= 1, P= 0.545										
0.070	1.716	0.957-3.076	82.115	< 0.001	5	27.957	6	Good	Quality of the studies	
0.004	2.107	1.264-3.510	44.465	0.145	3	5.402	4	Satisfactory		
Test for subgroup differences: Q= 0.270, df(Q)= 1, P= 0.604										
0.280	1.392	0.764-2.538	66.217	0.031	3	8.880	4	≤ 4 W	Time point of postpartum depression	
0.004	2.312	1.310-4.080	78.412	<0.0001	5	23.161	6	> 4 W		
Test for subgroup differences: Q= 1.447, df(Q)= 1, P= 0.229										
0.017	2.604	1.188-5.705	71.653	0.014	3	10.583	4	≤ 2 W	Time point of postpartum anemia	
0.061	1.548	0.980-2.444	72.569	0.003	5	18.228	6	> 2 W		
Test for subgroup differences: Q= 1.261, df(Q)= 1, P= 0.261										
0.312	1.167	0.866-1.572	32.994	0.201	4	5.970	5	≤ 4 W	Time point of postpartum depression	Anemia during pregnancy^*^
0.112	1.324	0.936-1.872	57.667	0.094	2	4.724	3	> 4 W		
Test for subgroup differences: Q= 0.294, df(Q)= 1, P= 0.588										

N; Number, RR; relative risk, W; week, **CI; Confidence interval, Q; Q test for heterogeneity, df; degrees of freedom, and I**^2^**; I square.**

* We could not do other subgroup analysis

**Figure 4:**
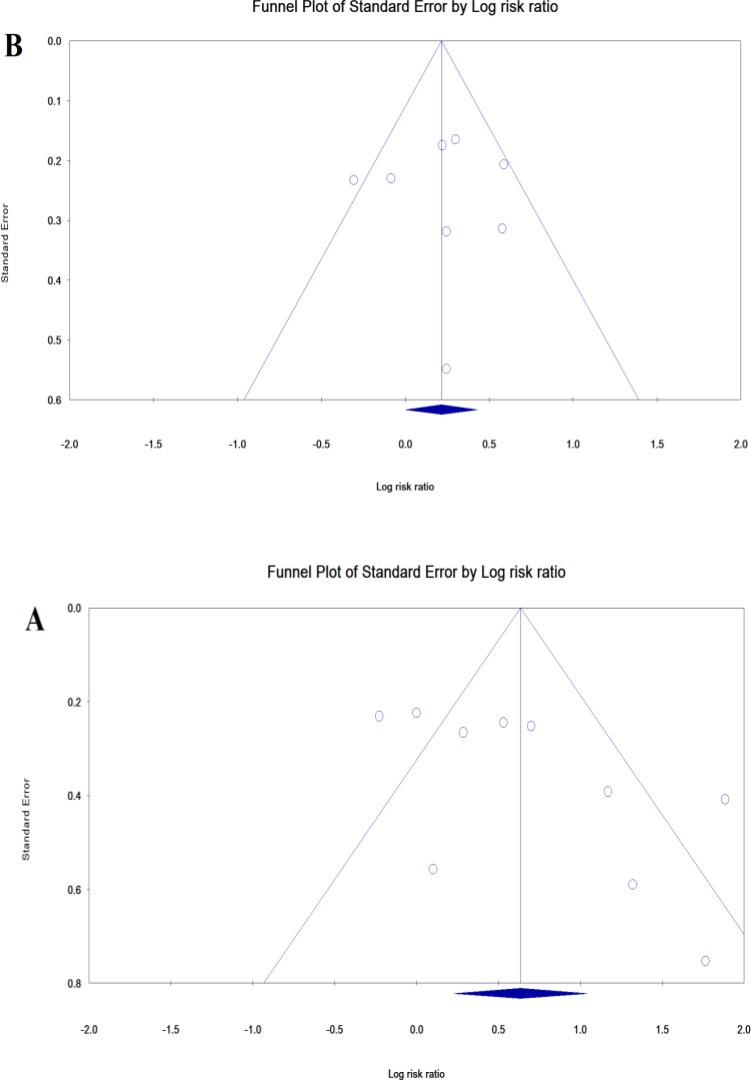
Publication bias for studies of postpartum anemia (A) and anemia during pregnancy (B) on postpartum depression

**Publication bias** **: **Publication bias for postpartum anemia and PPD was shown as a funnel plot, and p-value for Egger and Begg’s tests were 0.0528 and 0.0736, respectively, indicating that publication bias did not affect the results of the studies. Egger (p=0.710) and Begg’s (p=0.956) tests were not significant for anemia during pregnancy and PPD (figure 4).

**Grading of evidence:** According to GRADE summaries, we considered the quality of the evidence to be moderate for all outcomes. GRADE summaries were provided in table 3.

**Table 3:** GRADE assessment of confidence in effect estimates

**Outcome**	**Risk of bias**	**Consistency**	**Directness**	**Precision**	**Publication bias**	**Quality**
postpartum anemia on PPD	No serious limitations	Serious limitations^a^	No serious limitations	No serious limitations	No serious limitations	Moderate
Anemia during pregnancy on PPD	No serious limitations	Serious limitations^b^	No serious limitations	No serious limitations	No serious limitations	Moderate

*GRADE;* Grading of Recommendations Assessment, Development and Evaluation

a Considerable heterogeneity: I^2^ = 74.62%

b Moderate heterogeneity: I^2^ = 36.42%

## Discussion

The present study is the first systematic review and meta-analysis of the association between anemia and PPD. The results of the association between anemia and PPD were not similar in different studies; in the studies of Armony et al. (2012) (22), Eckerdal et al. (2016) (33) and Paterson et al. (1994) (34), this association was not significant, but it was significant in the studies of Akbari et al. (2008) (32), Alharbi et al. (2014) (23) and Corwin et al. (2003) (21). Based on the results of this study, PPD was significantly higher in anemic women versus non-anemic women with RR= 1.887 and P=0.002. Moreover, the association between anemia during pregnancy and PPD was significant (RR= 1.39 [1.15-1.68], P=0.001). Although these are primary studies in this field, the final decision on whether or not anemia has been associated with depression in women during pregnancy and after pregnancy has not been made. In this study, according to the systematic review of all the documents and their combination by meta-analysis, this association was investigated, which indicated the existence of the association. Subgroup analysis to find out the cause of heterogeneity revealed that quality of studies, study design, timing of depression and anemia are not the influenced factors. Some studies indicated the depression during pregnancy and PPD were associated with thyroid disorders and gestational diabetes (37-39). Anemia during pregnancy and after pregnancy significantly increased the risk of postpartum depression. Hemoglobin decline may change the function of neurotransmitters and subsequently alter the cellular, oxidative and thyroid hormones metabolism. In addition, the reduction of inflammatory cytokines, such as interleukin 2, as causative agents for anemia, can be an influencing factor in depression. Therefore, anemia during pregnancy and after pregnancy may be one of the causes of depression by altering inflammatory cytokines (21). However, according to the findings of this study, PPD was significantly higher in anemic women versus non-anemic women, and there was a significant association between anemia during pregnancy and PPD. Although, the main causes of PPD in clinical sciences are not yet known (21, 37-40). In another study, the symptoms of depression ranged from 48 hours after pregnancy to 32 weeks after pregnancy were examined in 821 women with low serum ferritin levels. They showed that if serum ferritin was about 1 μg, the risk of PPD would increase by 3.98 times (41). In another study, bleeding more than 1000 ml after childbirth increased the chance of anemia and increased the risk of depression by 2.1 times (34). 

In other studies, fatigue has been mentioned as one of the causes of depression; fatigue indicates a decrease in body energy levels and, consequently, the level of activity decreases to reduce energy consumption and to achieve balance. Increasing metabolic needs can explain the fatigue associated with pregnancy and postpartum period, and in this case, the higher the fatigue of the mother, the greater the likelihood of depression (42, 43). In investigation of the role of hormones in depression in pregnant and postpartum women, no major physiological hormone differences have been observed in women with PPD (21). In addition to physiological changes, sexual abuse, history of neurodegenerative disease or depression among relatives, especially close relatives, neglect from the spouse and relatives of the pregnant woman, fear of childbirth, marital problems, especially emotional and economic problems, low age of mother, marriage forced by parents, disinterest toward spouse and unwanted pregnancy can be other causes of depression during pregnancy and after childbirth (21, 44, 45). Publication bias for postpartum anemia on PPD studies has been shown as a funnel graph, and p-value for Egger’s and Begg’s tests were 0.06 and 0.08, respectively, indicating that publication bias did not affect the results of the studies. For anemia during pregnancy and PPD, Egger (0.826) and Begg’s (0.999) tests were not significant. It is assumed that the observed differences are due to different sampling and also the difference in the measured parameters in different societies. According to the World Health Organization's motto in 2017 (Depression: Let's talk), depression is a common emotional disorder and should be considered as a global health problem in all countries and societies. Family quality of life, early diagnosis of the disease in early weeks after delivery and early treatment are important (20, 46). Therefore, the study and treatment of anemia during pregnancy and after pregnancy can be an important preventive and therapeutic measure.

One of the limitations of this study is the lack of access to the Gray literature of different countries for the collection of more basic studies and more detailed examination.

In Conclusion, Meta-analysis results showed a significant association between anemia (during pregnancy and postpartum) and PPD. Therefore, prevention, identification and treatment of anemia in pregnant women seem necessary.

Appendix 1: PubMed search strategy

1. Anemia [Title, Abstract]
2. Anaemia [Title, Abstract]
3. Hemoglobin [Title, Abstract]
4. Ferritin [Title, Abstract]
5. Pregnancy [Title, Abstract]
6. Pregnant Woman [Title, Abstract]
7. Prenatal Care [Title, Abstract]
8. Complications of Pregnancy [Title, Abstract]
9. Depression [Title, Abstract]
10. Postpartum Depression [Title, Abstract]
11. Mental Disorders [Title, Abstract]
12. Mental Health [Title, Abstract]
13. 1 OR 2 OR 3 OR 4
14. 5 OR 6 OR 7 OR 8
15. 9 OR 10 OR 11 OR 12
16. 13 AND 14
17. 13 AND 15
18. 15 AND 16
